# Complete mitochondrial genomes of Karchaev goat (*Capra hircus*)

**DOI:** 10.1080/23802359.2020.1831988

**Published:** 2020-10-27

**Authors:** Andrey N. Rodionov, Arsen V. Dotsev, Oleg Y. Fomenko, Neckruz F. Bakoev, Tatiana E. Deniskova, Alexey V. Shakhin, Vugar A. Bagirov, Elisabeth Kunz, Ivica Medugorac, Stefan Krebs, Gottfried Brem, Natalia A. Zinovieva

**Affiliations:** a L.K. Ernst Federal Science Center for Animal Husbandry, Podolsk, Russia; b Department of Veterinary Sciences, Ludwig-Maximilians-University, Munich, Germany; c Gene Center, Ludwig-Maximilians-University, Munich, Germany; d Institute of Animal Breeding and Genetics, Vienna, Austria

**Keywords:** Domestic goats, Caprinae, mitogenome, haplogroup

## Abstract

Karachaev goat (*Capra hircus*) is a local breed from North-Caucasus region, Russia. Here we present complete mitochondrial genome of Karachaev goat from the republic of Karachaevo-Cherkessia, Russia. The length of the studied sequence was 16,624 bp in size. It was shown that the studied specimen belonged to haplogroup A.

Karachaev goat (*Capra hircus*) is mainly raised in North-Caucasus region of Russia. It is a meat-purpose breed, which is well adapted to the local environment and able to use high mountain pastures (Mamontova et al. 2012). Its habitat is closely related to wild goats – Caucasian tur (*Capra caucasica*) making possible uncontrolled interbreeding between the two species (Klenovitsky et al. 2018). In our work, we describe complete mitochondrial genome of Karachaev goat and determine its phylogenetic placement within genus Capra.

For this study, the muscle tissue of Karachaev goat was collected in the republic of Karachaevo-Cherkessia, Russia and deposited in the biobank at the L.K. Ernst Federal Science Center for Animal Husbandry, Russia. Genomic DNA was extracted using a Nexttec column (Nexttec Biotechnology GmbH, Germany). The sequencing of mitochondrial DNA was performed using NGS technology on a HiSeq 1500 (Illumina) and the obtained reads were assembled with bwa mem algorithm in bwa 0.7.17 software (Li and Durbin 2010). To improve accuracy of the newly assembled genomes, in terms of indels, the hypervariable part of the control region (D-loop) was additionally sequenced by Sanger technology using forward and reverse sequencing primers (F-5′-ATACCAGCAGCTAGCACCATT-3′ and R-5′-GGCATTTTCAGTGCCTTGCTT-3′). The complete mitogenome of Karachaev goat was deposited in GenBank with accession number: MT396986.

Phylogenetic analysis based on the Bayesian inference method was performed in MrBayes 3.2.6 (Ronquist et al. 2012) on the concatenated datasets of 13 protein-coding genes (PCGs) and 2 rRNAs of Karachaev goat, 11 worldwide goat breeds and five wild bezoar goats (*Capra aegagrus*) which belonged to different haplogroups (Colli et al. 2015). Caucasian tur (*Capra caucasica*) sequence (Hassanin et al. 2012) was taken as an outgroup. The DNA sequences were aligned using the MUSCLE algorithm (Edgar 2004), as implemented in R package msa (Bodenhofer et al. 2015). MITOS WebServer (Bernt et al. 2013) was used to annotate the mitochondrial genomes. PartitionFinder 2 (Lanfear et al. 2017) was used to select the best partitioning scheme and evolutionary models.

The whole mitochondrial genome of Karachaev goat was 16,624 bp in length and had gene arrangement pattern similar to the typical vertebrate mitochondrial genome, containing 13 PCGs, 22 tRNA genes, 2 rRNA genes, and a non-coding region. An overall nucleotide composition was: A = 5572 (33.52%), T = 4546 (27.35%), C = 4325 (26.02%), G = 2181 (13.12%). The AT-skew was positive (0.101 and 0.102) and GC-skew was negative (−0.330 and −0.329). Genetic analysis and phylogenetic reconstruction (Figure 1) revealed that the Karachaev goat sequence belonged to haplogroup A.

**Figure 1.**
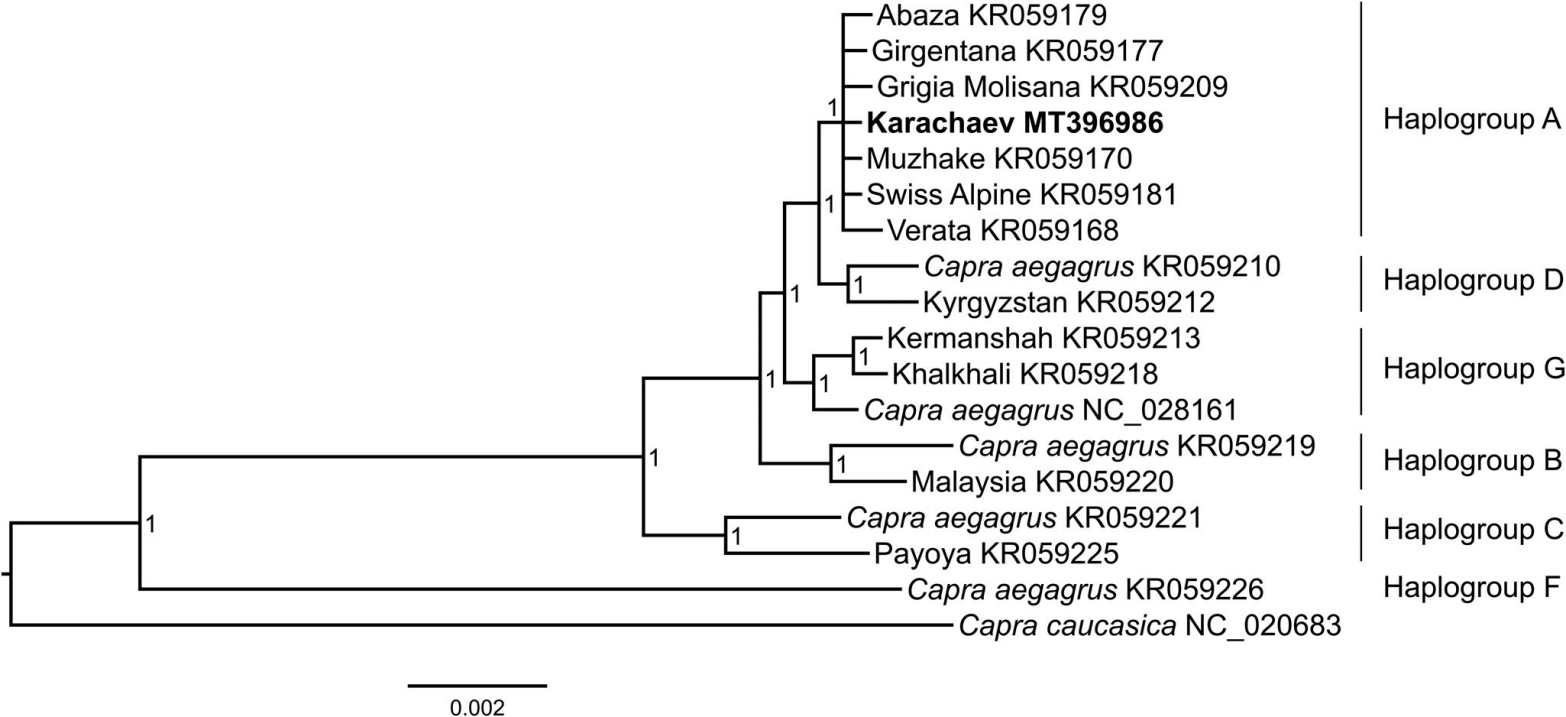
Bayesian tree based on the concatenated nucleotide sequences of 13 mitochondrial PCGs and two mRNA indicating phylogenetic relationship of Karachaev goat (in bold) with worldwide goat breeds and Bezoar goat (*Capra aegagrus*). Node numbers show posterior probability values. GenBank accession numbers are given with species names.

In this study, we provided the mitogenome sequence of Karachaev goat, which will be important in further taxonomic classification, and as well as in implementing conservation and breeding strategies.

## Data Availability

The data that support the findings of this study are openly available in ‘NCBI’ at https://www.ncbi.nlm.nih.gov/, reference number: MT396986.
